# Carbon dioxide pneumothorax following retroperitoneal laparoscopic partial nephrectomy: a case report and literature review

**DOI:** 10.1186/s12871-018-0662-x

**Published:** 2018-12-22

**Authors:** Qiongfang Wu, Hong Zhang

**Affiliations:** Departments of Anesthesiology and Critical Care Medicine, Peking University First Hospital, Beijing, 100034 China

**Keywords:** Carbon dioxide, Pneumothorax, Laparoscopy, Case report

## Abstract

**Background:**

Laparoscopy has many advantages when used to assist surgery. However, pneumothorax, as a rare but potentially life-threatening complication, it requires rapid recognition and treatment. CO_2_ pneumothorax may be distinct from air pneumothorax. Here we present a case with unexpected large and symptomatic CO_2_ pneumothorax and treated successfully in a conservative way.

**Case presentation:**

A 27-year-old woman who was scheduled a laparoscopic partial nephrectomy received general anesthesia. At the end of surgery, she waked up and got spontaneous breathing. However, she developed a sudden fall in SpO_2_ (approximately 30%) and blood pressure with subsequent unconsciousness after switching mechanical ventilation to spontaneous mode. With immediate manual ventilation, SpO_2_ and blood pressure recovered simultaneously and the patient regained consciousness. Point-of-care chest X-ray revealed a large, right pneumothorax occupying 70% of the hemi-thorax. Without chest drainage, she was extubated in the operating room and treated with supplemental facial mask oxygen therapy in PACU. On the postoperative 5th day, she was discharged without any further complication.

**Conclusion:**

Retroperitoneal laparoscopic surgeries are likely to bring about severe capno-thorax, which could be absorbed rapidly. Chest X-ray could be used to assist diagnosis but point-of-care transthoracic ultrasound is recommended. Even severe capno-thorax could be treated conservatively. This case highlights the awareness and therapeutic choice of noninvasive management for capno-thorax.

## Background

Laparoscopic surgeries have been widely performed because they have so many advantages as less invasiveness, less postoperative pain, shorter hospital stay and better cosmesis [1]. However, they may also cause severe complications like pneumothorax, pneumomediastinum and subcutaneous emphysema [2]. Laparoscopic partial nephrectomy, which could be performed through insufflating carbon dioxide (CO_2_) in either the retroperitoneal or peritoneal space to achieve a better view, has been widely used for surgical treatment for T1 stage renal tumor. Pneumothorax is a recognized complication of laparoscopic surgery [3, 4]. Although the incidence of pneumothorax in laparoscopic renal surgery is less than 1%, it’s potentially serious and life-threatening [5], requiring rapid recognition and treatment. For patients with large pneumothorax or obvious symptoms, the common practice is to place a chest tube for drainage. However, CO_2_ pneumothorax is distinct from air pneumothorax in that CO_2_ is highly soluble in blood and could be absorbed rapidly [6, 7]. Here, we present a case with an unexpected large volume of CO_2_ pneumothorax and severe symptoms, which was managed conservatively.

## Case presentation

A 27-year-old woman (height 165 cm; weight 92 kg) was admitted to hospital for management of a 1.8 cm × 1.3 cm × 1.7 cm renal mass in the lower pole of the right kidney (examined by MRI) and scheduled for laparoscopic partial nephrectomy under general anesthesia. The patient mentioned she had no other medical history but congenital asymptomatic platybasia. The laboratory examinations were normal except uric acid 425 μmol/L (the normal range is 90–360 μmol/L). There was no abnormity for electrocardiogram (ECG) or chest X-ray.

On the operating day, the patient entered the operating room without premedication. ECG, SpO_2_, end-tidal carbon dioxide pressure (PetCO_2_) and bispectral index (BIS) were monitored. A 20G catheter was inserted into her left radial artery to ensure real-time blood pressure monitoring. Anesthesia was induced with remifentanil (target-controlled infusion at effect-site concentration of 3 ng/mL), 150 mg propofol and 50 mg rocuronium. A 7 mm ID endotracheal tube was intubated with an insertion distance of 21 cm at the incisors. The patient was ventilated with volume controlled ventilation mode (setting tidal volume at 500 ml, respiratory rate at 12 times/min, inspiration and expiration ratio at 1:2) and was placed in the left lateral decubitus position. Before pneumoperitoneum, airway pressure was controlled within 20 cmH_2_O and PetCO_2_ was controlled between 31 and 35 mmHg. Anesthesia was maintained with intravenous remifentanil (target-controlled infusion at effect-site level of 2–3 ng/mL), propofol (constant infusion), and 60% nitrous oxide balanced with oxygen. Sufentanil (totally 20 μg) and cisatracurium (totally 2 mg) was intermittently injected intravenously and the infusion speed of propofol was adjusted according to BIS within the range of 40 to 60.

**Fig. 1:**
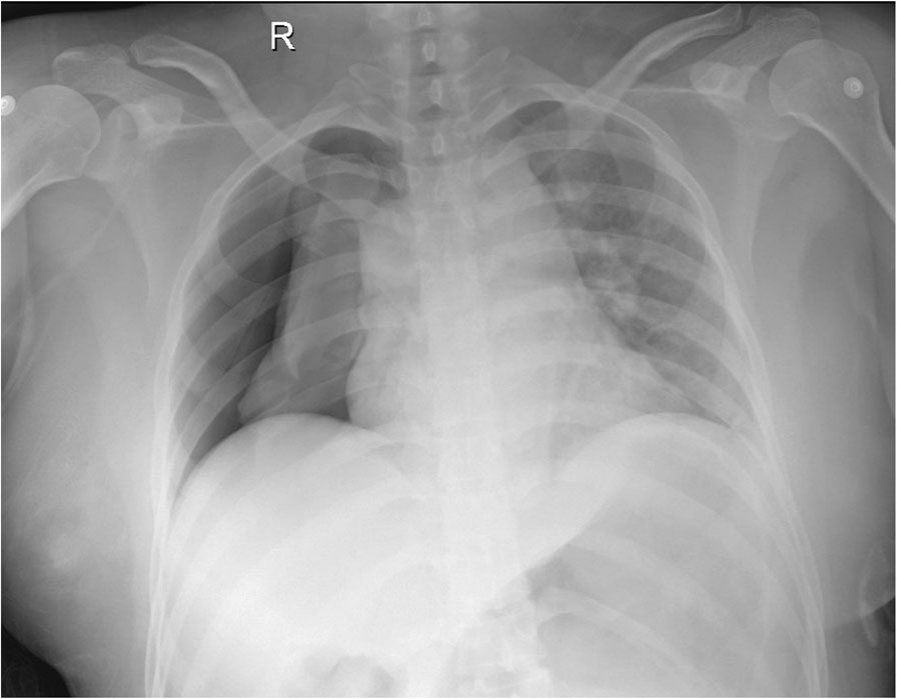
Chest X-ray in the operating room

The procedure was uneventful though there was an episode. After finishing the first trocar portal, a balloon, made from a sutured latex glove, was used to inflate and dilate retroperitoneum cavity. But the balloon was ruptured during inflation and the suture on the balloon was left in retroperitoneum space. Finally, the suture was found and removed after a period of searching with the help of laparoscopy. Procedure was performed via 3 successfully established trocar portals. The retroperitoneal space was hydrostatically dilated and CO_2_ was insufflated to a pressure of 14 mmHg. During the procedure, the airway pressure increased to 30 cmH_2_O and PetCO_2_ was elevated to 41 mmHg. SpO_2_ remained 100%. We adjusted tidal volume to 550 ml and respiratory rate to 13 times/min. At the end of the surgery, the retroperitoneal carbon dioxide was retreated and airway pressure decreased to 23–24 cmH_2_O. Operative duration was almost 90 min, during which the hemodynamic parameters were stable. We stopped all intravenous anesthetics and changed the gas supply to 100% O_2_ with a flow rate of 5 L/min. The patient woke up quickly and could respond to instructions. Then the patient was placed to supine position. Mechanical ventilation was ceased and the patient had spontaneous breathing. Antagonists of muscle relaxant (neostigmine 2 mg plus atropine 1 mg) were given. Two minutes after the stop of mechanical ventilation, SpO_2_ decreased rapidly to 30% and blood pressure decreased from 120/79 mmHg to 93/65 mmHg. The patient was unconscious. We conducted manual ventilation immediately and then SpO_2_ returned to 85–90%. Meanwhile, blood pressure recovered. Immediate auscultation showed decreased breath sounds on the right side of chest and the left side was normal. Immediate arterial blood gas analysis showed PCO_2_ 75 mmHg, PO_2_ 83 mmHg. We woke up the patient and she could breathe spontaneously with better tidal volume. We extubated her endotracheal tube and provided oxygen via facemask. She was able to breathe without distress but felt right chest pain. SpO_2_ climbed to 91–93% gradually. Point-of-care chest X-ray was performed, demonstrating a large, right pneumothorax occupying 70% of the hemithorax (Fig. 1).

The patient was transferred to post-anesthesia care unit and stayed for 1 h with facemask oxygen inhalation (oxygen flow rate 5 L/min). Her vital signs were normal during the period and SpO_2_ returned to 100%. She felt right chest pain relieved a bit. Arterial blood gas was re-examined and all parameters were in normal range. The patient was later sent back to general ward.

**Fig. 2:**
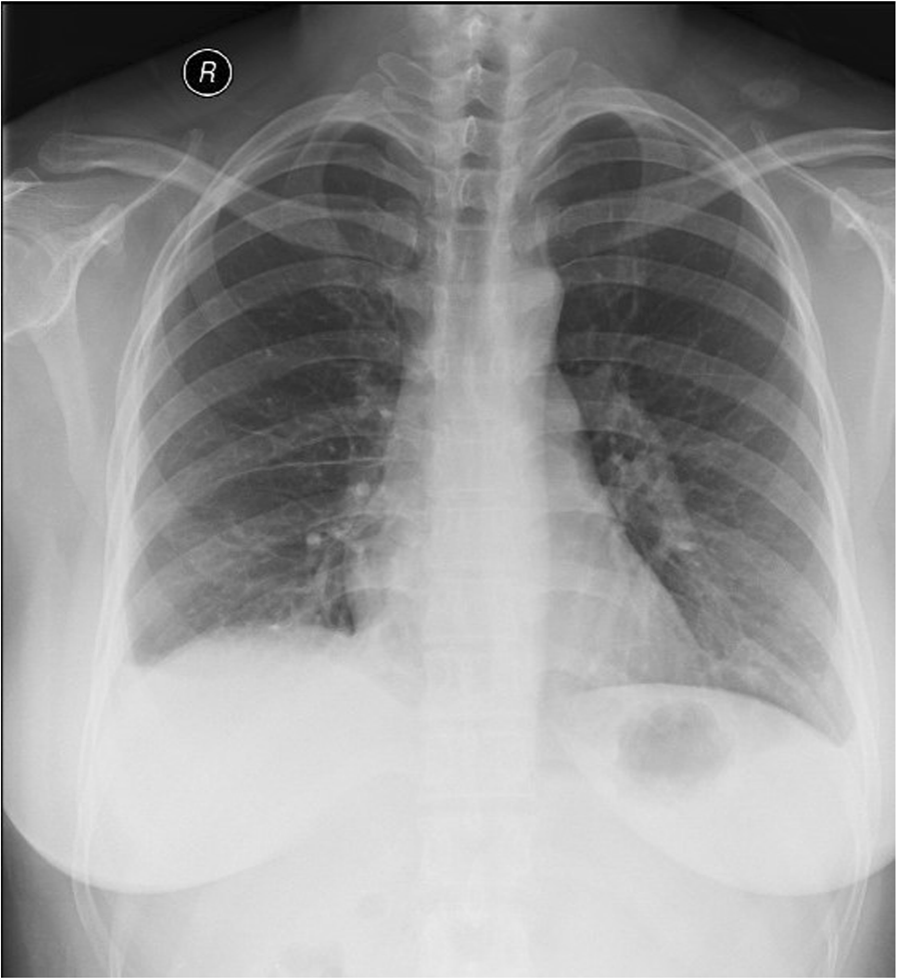
Chest X-ray on the first postoperative day

In general ward, respiratory and hemodynamic parameters of the patient remained stable during hospital stay. Chest X-ray taken on the first post-operative day revealed complete re-expansion of the right lung (Fig. 2). She claimed no chest pain and no other symptoms. The patient was discharged home on postoperative day 5.

## Discussion and conclusion

Large or symptomatic pneumothorax usually necessitates thoracocentesis or chest tube placement. But capno-thorax may warrant a different treatment. Table 1 summarized cases which reported moderate to large symptomatic capno-thorax while implemented conservative treatments. Different from these described above, in our case, the type of surgery route was retroperitoneal. The symptom was more severe (SpO_2_ 30%) and happened after gas deflation and before extubation. The patient was awake at first but fell into unconsciousness later. It’s more urgent and hard for us to diagnose and decide whether it was suitable to adopt an invasive method. Fortunately, the severe condition didn’t last for a long time. We also retrieved some cases in whom capno-thorax occurred in retroperitoneal urological surgeries. But most of these happened intraoperatively and implemented thoracocentesis or chest tube placement [8–11], or had confirmed diaphragm injury and recovered after converting to open surgery [12]. Although study showed CO_2_ absorption during laparoscopy did not depend on the route of surgery [13, 14], for retroperitoneal route, the restriction on working space and field of view may increase the risk of inadvertent organ damage.

**Table 1:** Summary of reports on adopting conservative treatments in patients with capno-thorax during laparoscopic surgeries

Reference	Type of surgery	Route of surgery	Time to discovery	Associated condition (the lowest SpO_2_)	Causes	Volume ^a^	Special treatment ^b^
Waterman et al. [3]	Upper pole partial nephroureterectomy	Transperitoneal	End of surgery	Unstable respiratory condition (93%)	Possible long surgery duration, high peritoneum pressure, unrecognized congenital defects	Moderate (unilateral)	Transient deflation
	Percutaneous endoscopic Cohen reimplant of ureters	Intravesical via extraperitoneal	Intraoperative	Unstable respiratory condition, subcutaneous emphysema (60%)		Large (left), small (right)	No
Park et al. [15]	Gastrectomy	Transperitoneal	Intraoperative	Unstable respiratory condition (94%)	Confirmed congenital diaphragm defect	Large (unilateral)	Transient deflation
Msezane et al. [28]	Partial nephrectomy	Transperitoneal	Intraoperative	Stable (unchanged)	Confirmed diaphragm injury	Large (unilateral)	Diaphragm laceration repaired
Karayiannakis et al. [29]	Cholecystectomy	Transperitoneal	Intraoperative	Unstable respiratory condition (92%)	Possible congenital diaphragm defect	Large (unilateral)	No
Mehran et al. [31]	Bariatric surgery	Transperitoneal	Intraoperative	Unstable cardiopulmonary condition (100%)	Unclear	Large (unilateral)	Transient deflation, peritoneum pressure reduced
Altarac et al. [32]	Ureterolysis	Transperitoneal	Intraoperative	Unstable respiratory condition, subcutaneous emphysema (unchanged)	Possibly diaphragm defect	NA (unilateral)	Converted to open surgery.
Kumar et al. [33]	Cholecystectomy	Transperitoneal	Intraoperative	Unstable cardiopulmonary condition (85%)	Unclear	apical (unilateral)	No
Bala et al. [34]	Cholecystectomy	Transperitoneal	Intraoperative	Unstable cardiopulmonary condition (85%)	Unclear	NA (unilateral)	No
Phillips el al. [35]	Hiatus hernia repair	Transperitoneal	10 intraoperative, 1 postoperative	Unstable cardiopulmonary condition (NA)	Surgical dissection	NA (2 bilateral)	NA

NA, not available

^a^All diagnosed by chest X-ray;

^b^Treatment of pneumothorax besides increased FiO_2_, improved ventilator settings and close monitoring

### Causes for pneumothorax

Pneumothorax during laparoscopic procedures has been reported and the possible causes are [3, 8–14]: First, barotrauma and rupture of bullae during mechanical ventilation or during central venous line placement may contribute to air pneumothorax. Second, unrecognized congenital defect such as diaphragmatic defects [15] or pleuroperitoneal fistulas could predispose patients to pneumothorax. Third, increased operative duration is likely to compel more CO_2_ absorption. Study showed that PetCO_2_ greater than 50 mmHg and operative time greater than 200 min were risk factors for pneumothorax [16]. Fourth, higher insufflation pressure likely leads to a large amount of CO_2_ absorption. Several cases have been reported to get pneumothorax during laparoscopic surgeries with an insufflation pressure of 12–15 mmHg [2, 17, 18] whereas insufflation pressure under 12 mmHg might still cause pneumothorax [19]; furthermore, rapid insufflation would allow the intracavitary pressure to increase suddenly, contributing to pneumothorax [11] because retroperitoneal space is not a true cavity and has lower compliance than the abdominal cavity. Fifth, it is also possible for CO_2_ to dissect into the pleural space along the vena cava or aorta [16]. Finally, pleural injury due to errant trocar placement or diaphragmatic injury possibly accounts for pneumothorax [4, 20].

In our case, the symptomatic pneumothorax was unanticipated as the procedure and anesthesia practice was uneventful. The patient was young and healthy. Operating duration and intraoperative PetCO_2_ were also acceptable. But the diaphragmatic defect cannot be excluded. Though operation on the lower pole of the kidney is less likely to injure diaphragm, the procedure to explore the lost suture might injure it unconsciously. Besides, CO_2_ could also dissect into the pleural space along the vena cava. All these possible reasons suggest not air but CO_2_ pneumothorax.

### Clinical manifestations and diagnosis

Pneumothorax could bring out pulmonary atelectasis, elevate inspiratory airway pressure, PetCO_2_ as well as PaCO_2_ and decrease breath sounds and blood pressure. Hypotension occurs due to the decreased venous return and cardiac output. However, in this case, these changes were not obvious during procedure but appeared after the termination of mechanical ventilation. Possible reasons are that respiratory depression after the cessation of mechanical ventilation combined with pneumothorax exaggerated the clinical manifestation.

Chest computer tomography scan is the gold standard for diagnosis of pneumothorax [21, 22]. Chest X-ray is usually as the initial tool to detect the potential cases. All the cases list above was diagnosed by Chest X-ray. However, it has been demonstrated that point-of-care transthoracic ultrasound, a cheaper, nonradiative and timely tool, is more accurate than chest X-ray with 81% sensitivity and 100% specificity [23, 24]. Anterior absent lung sliding plus A lines plus lung point indicated pneumothorax [23]. If lung is totally collapsed, lung point isn’t visualized; lung point on the mid axillary line differentiates large and small pneumothorax, coinciding with a cut-off set at 15% of lung collapse [25], and could guide decision-making in treatments. This technique is extremely important for anesthesiologists to practice in the operating room. However, we didn’t perform ultrasound scan because of inexperience.

### Practice taken to alleviate carbon dioxide pneumothorax

The treatment can vary depending on the causes and severity of the pneumothorax. FiO_2_ should be increased and N_2_O supply should be stopped. CO_2_ insufflation could be reduced or discontinued. Endotracheal intubation, hyperventilation and higher positive end-expiratory pressure should be maintained [26].

Traditionally, a chest tube could be placed if a large pneumothorax or consequent cardiovascular or respiratory collapse is diagnosed. But capno-thorax may warrant a different treatment strategy. The solubility of CO_2_ is 20 times more than nitrogen and has an increased diffusion coefficient compared to air, which is composed mostly of nitrogen and oxygen. Higher solubility of a gas means more molecules can diffuse across a membrane in a given time. Experimental and clinical evidence demonstrated that resolution of capno-thorax can be more rapid than air pneumothorax, and usually complete re-expansion of the compressed lung could be achieved within several hours [15, 27–29], even within 30–60 min [30].We didn’t implement thoracentesis or place a chest tube but provided oxygen supply and intensive care because it is found that the probability of pneumothorax caused by CO_2_ is higher than that by air, patient’s condition was getting better and the source of CO_2_ was no longer feeding the retroperitoneum. The woman returned to normal state within 1 h although chest pain disappeared completely the next day.

In conclusion, pneumothorax, although rare, is a serious complication of laparoscopic nephrectomy. Anesthesiologists, when facing this crisis, should recognize pneumothorax right away. Point-of-care transthoracic ultrasound should be recommended to diagnose. Different from air pneumothorax, CO_2_ pneumothorax, even with a large volume, can be resolved spontaneously without invasive management.

## Abbreviations

BIS: bispectral index
CO_2_: carbon dioxide
CXR: chest X-ray
ECG: electrocardiogram
FiO_2_: fraction of inspired oxygen
MRI: magnetic resonance imaging
N_2_O: nitrous oxide
O_2_: oxygen
PCO_2_: carbon dioxide partial pressure
PetCO_2_: end-tidal carbon dioxide pressure
PO_2_: oxygen partial pressure
SpO_2_: pulse oxygen saturation
